# Exploring the Impact of Gaming Habits on Sleep Patterns Among Young Adults in Saudi Arabia: A Cross-Sectional Study

**DOI:** 10.7759/cureus.56224

**Published:** 2024-03-15

**Authors:** Saba Firdos, Sarah Al-Omar, Farha Aldossary, Taif Alshamrani, Mashael Alhussain, Taif Al-Otaibi, Ibraheem Alhusain

**Affiliations:** 1 Clinical Neuroscience, King Faisal University, Al-Ahsa, SAU

**Keywords:** obesity, cross-sectional study, health outcomes, young adults, sleep patterns, video games

## Abstract

Background

Video gaming is increasingly popular among young adults, potentially affecting health and daily routines, especially sleep patterns. In Al-Ahsa, Saudi Arabia, the impact of video gaming on sleep and health among young adults is not well understood. This study aims to explore this relationship, focusing on how video gaming habits influence sleep patterns and associated health outcomes.

Methods

This cross-sectional survey utilized an adapted online questionnaire to gather data on demographics, video gaming habits, sleep patterns, and body mass index from young adults in Al-Ahsa. Statistical analysis, including descriptive statistics, chi-square tests, and multivariable logistic regression, was applied to examine the associations between video gaming habits and sleep sufficiency.

Results

The study included 302 participants, including 165 (54.6%) females. A majority reported playing video games for less than one hour daily (36.1%), with 82.5% preferring online gaming. Notably, 54.3% of participants slept more than six hours nightly, yet challenges with sleep initiation were evident, as 48.0% went to bed past midnight. Multivariable logistic regression highlighted significant factors affecting sleep sufficiency: participants who played games after completing tasks had 80% lower odds of sleep insufficiency compared to those who played before tasks. Overweight participants were found to be 7.7 times more likely to experience sleep insufficiency compared to their underweight peers.

Conclusion

The study underscores a complex relationship between video gaming habits and sleep patterns among young adults in Al-Ahsa, with significant health implications. It suggests the necessity for interventions promoting balanced gaming habits and improved sleep hygiene to mitigate adverse health outcomes.

## Introduction

The exploration of the relationship between video game use and its impacts on health and sleep patterns has garnered significant attention within the scientific community, especially concerning young adults [1]. This heightened interest is partly due to the pervasive nature of video gaming in modern society, coupled with emerging concerns over its potential negative health outcomes [2]. The background of this study situates within this broader discourse, particularly focusing on the Al-Ahsa region of Saudi Arabia, where gaming is a prevalent activity among young adults.

Video games have evolved from simple entertainment to complex virtual environments where individuals, especially young adults, spend a considerable portion of their daily lives. This evolution has prompted researchers to examine the multifaceted effects of video gaming on physical, psychological, and behavioral health [3]. Studies have shown that while video gaming can improve certain cognitive skills and provide social benefits through online interactions, excessive gaming can lead to sleep disturbances, psychological distress, and physical health problems such as obesity and cardiovascular risks [4].

Sleep, a fundamental component of overall health, is particularly affected by video gaming. The stimulating nature of video games, especially when played before bedtime, can interfere with the body’s natural sleep mechanisms, leading to difficulties falling asleep, decreased sleep quality, and reduced sleep duration [5]. These disruptions in sleep patterns are of particular concern among young adults, a group known to be at risk for insufficient sleep due to various behavioral and lifestyle factors, including the intensive use of digital media.

Moreover, the health implications of altered sleep patterns due to video gaming extend beyond immediate sleep disturbances. Insufficient or poor-quality sleep has been linked to various long-term health issues, including obesity, hypertension, and insulin resistance [6]. These conditions contribute to a heightened risk of more severe health complications, such as type 2 diabetes and cardiovascular diseases, emphasizing the need for a deeper understanding of the relationship between video game use, sleep, and health outcomes.

Given this context, the study aims to shed light on these associations within the Al-Ahsa region, an area characterized by its unique demographic profile and cultural context. By focusing on young adults in this region, the research seeks to contribute to the existing literature by offering insights into how video gaming behaviors influence sleep patterns and health, potentially guiding future interventions and public health strategies.

## Materials and methods

Study design and setting

This research was framed as a cross-sectional study aimed at exploring the impact of video game usage on sleep patterns and health outcomes among young adults in the Al-Ahsa region of Saudi Arabia. The focus was specifically on university students and young adults within this geographical area, recognizing the prevalence of video game use within this demographic and its potential implications for health and sleep quality.

Objectives

The study was driven by two primary objectives: firstly, to elucidate the relationship between video game habits and sleep patterns among young adults in Al-Ahsa, and secondly, to investigate how these sleep patterns may, in turn, affect the health of this population. These objectives were grounded in the hypothesis that the prevalent video game usage among young adults in Al-Ahsa could lead to sleep disturbances and subsequent health issues, including hypertension, insulin resistance, and obesity.

Study participants

The study targeted a broad spectrum of young adults and university students residing in the Al-Ahsa region. The inclusion criteria were set to encompass individuals within this demographic to capture a comprehensive overview of gaming habits and their health implications.

Sample size determination and sampling procedure

The sample size was determined to be 284 individuals, based on the Richard Geiger equation, taking into account a 5% margin of error, a confidence level of 95%, and an assumed response distribution of 50% within the target population estimated at 50,000. Participants were recruited through a random sampling strategy, employing an electronic questionnaire distributed via Google Forms (Google LLC, Mountain View, CA, USA). Informed consent was obtained from all participants before inclusion in the study, ensuring ethical standards were upheld.

Data collection and instrumentation

Data collection was carried out using an online survey questionnaire, which was meticulously developed following a review of related literature and consultation with subject matter experts to ensure content validity. The questionnaire, designed to gather comprehensive data on video gaming behaviors, sleep patterns, and health indicators, was adapted from a previous study and further refined through a pilot study to confirm its reliability [7]. The survey encompassed a range of questions covering demographic information, gaming habits, sleep quality, and health-related questions, designed to capture both quantitative and qualitative aspects of the participants’ experiences and behaviors. The data collected through this survey were then rigorously analyzed, employing statistical methods suitable for the research questions at hand, with a particular focus on exploring the associations between video game use, sleep patterns, and health outcomes among the young adult population in Al-Ahsa.

Ethical considerations

This study adhered to strict ethical guidelines, securing approval from the Institutional Review Board of King Faisal University to ensure adherence to ethical research practices (approval number 2023-1664). Informed consent was obtained from all participants, emphasizing the voluntary nature of their involvement and the confidentiality of their responses. Participant information was anonymized to protect privacy, and ethical considerations were prioritized throughout the study to safeguard the welfare of all participants.

Statistical analysis

Statistical analyses were performed using IBM SPSS Statistics for Windows, Version 25.0 (Released 2017; IBM Corp., Armonk, NY, USA). Descriptive statistics, including means, standard deviations, and frequencies, were employed to summarize demographic data and gaming habits. Chi-square tests were utilized to examine associations between categorical variables. A multivariable logistic regression analysis was conducted to assess the impact of video gaming on sleep patterns and health outcomes, controlling for potential confounders. A p-value of less than 0.05 was considered statistically significant, ensuring the robustness of the findings.

## Results

Demographic analysis

The demographic analysis of the study participants, totaling 302, revealed a distribution across various age groups. Specifically, 123 individuals were aged between 23 and 25 years (40.7%), 88 individuals between 20 and 22 years (29.1%), and 91 individuals under 20 years (30.1%). Females constituted 165 of the sample (54.6%), while males accounted for 137 (45.4%). The majority of respondents, 287 individuals, were Saudi nationals (95%), predominantly from the Eastern Province, with 263 individuals (87.1%). Regarding marital status, 194 were single (64.2%), 92 were married (30.5%), and 16 were divorced (5.3%). Among the participants, 202 were university students (66.9%). The body mass index categories showed 137 individuals of normal weight (45.4%), 58 overweight (19.2%), 24 obese (7.9%), and 11 underweight (3.6%) (Table 1).

**Table 1:** Demographic characteristics of participants (N = 302)

Variable	Category	Frequency	Percent
Age group (years)	Under 20	91	30.1%
	20–22	88	29.1%
	23–25	123	40.7%
Gender	Male	137	45.4%
	Female	165	54.6%
Nationality	Saudi	287	95.0%
	Non-Saudi	15	5.0%
Region	Eastern Province	263	87.1%
	Western Province	10	3.3%
	Northern Province	11	3.6%
	Central Province	14	4.6%
	Southern Province	4	1.3%
Marital status	Single	194	64.2%
	Married	92	30.5%
	Divorced	16	5.3%
Student status	No	100	33.1%
	Yes	202	66.9%
Body mass index category	Underweight	11	4.8%
	Normal weight	137	59.6%
	Overweight	58	25.2%
	Obese	24	10.4%

Examination of gaming habits

The examination of gaming habits among the participants, totaling 302, illustrates distinct patterns in daily gaming duration, timing of gaming sessions, preference for gaming type, and variations in gaming time. A plurality of participants, 109 individuals, reported playing video games for less than one hour daily (36.1%), closely followed by 105 individuals playing between one and three hours (34.8%), and a significant portion, 88 individuals, engaged in more than three hours of gaming (29.1%). Regarding the timing of gaming sessions, the largest group, 100 individuals, preferred playing during break time (33.1%), with others, 76 individuals, playing after completing tasks (25.2%), 90 individuals at any time without a specific preference (29.8%), and a smaller fraction, 36 individuals, before completing tasks (11.9%).

A substantial majority of respondents favored online gaming, 249 individuals (82.5%), over offline gaming, 53 individuals (17.5%), indicating a significant preference for interactive gaming experiences. Additionally, there was a noticeable difference in the variation of gaming time, with more participants, 183 individuals, gaming extensively on weekends (60.6%) compared to weekdays, 119 (39.4%) individuals (Table 2).

**Table 2:** Distribution of gaming habits among participants (N = 302)

Variable	Category	Frequency	Percent
Daily gaming duration	Less than one hour	109	36.1%
	One to three hours	105	34.8%
	More than three hours	88	29.1%
Timing of gaming sessions	Before completing tasks	36	11.9%
	After completing tasks	76	25.2%
	During break time	100	33.1%
	Timing does not matter	90	29.8%
Gaming type preference	Offline	53	17.5%
	Online	249	82.5%
Variation in gaming time	More on weekdays	119	39.4%
	More on weekends	183	60.6%

Assessment of sleep patterns

The assessment of sleep patterns among the participants, with a total of 302, reveals insights into total sleep duration, sleep initiation time, morning wake-up times, and the duration it takes for individuals to fall asleep (Table 3). A slight majority of the participants, 164 individuals, reported a total sleep duration of more than six hours (54.3%), while a significant portion, 138 individuals, experienced less than six hours of sleep (45.7%). In terms of sleep initiation, a narrow majority, 157 individuals, managed to fall asleep before midnight (52.0%), compared to those who went to bed past midnight, 145 individuals (48.0%).

**Table 3:** Summary of sleep patterns among participants (N = 302)

Variable	Category	Frequency	Percent
Total sleep duration	More than six hours	164	54.3%
	Less than six hours	138	45.7%
Sleep initiation time	Before midnight	157	52.0%
	Past midnight	145	48.0%
Morning wake-up time	Before 7 am	128	42.4%
	7 am to 9 am	107	35.4%
	After 9 am	67	22.2%
Time to fall asleep	Thirty minutes	118	39.1%
	One hour to two hours	130	43.0%
	More than three hours	54	17.9%

Analysis of factors associated with sleep insufficiency

The analysis of factors associated with sleep sufficiency among participants revealed various statistically significant relationships and trends. Regarding daily gaming duration, participants who gamed for less than one hour reported a higher frequency of insufficient sleep (63.3%, p = 0.02) compared to those gaming for one to three hours (54.3%) and more than three hours (43.2%). The timing of gaming sessions was significantly associated with sleep sufficiency; participants gaming before completing tasks reported the highest frequency of insufficient sleep (86.1%, p < 0.01), followed by gaming after completing tasks (53.9%), during break time (47.0%), and when timing did not matter (50.0%).

A notable finding was in the body mass index category, where overweight participants experienced a higher frequency of insufficient sleep (67.2%, p < 0.01) compared to those with normal weight (53.3%) and obese participants (29.2%). The preference for online gaming was also associated with higher rates of insufficient sleep (57.0%, p = 0.04) compared to offline gaming (41.5%).

Additionally, gaming time variation showed significant differences; participants gaming more on weekends reported a higher frequency of insufficient sleep (59.6%, p = 0.02) compared to those gaming more on weekdays (46.2%). Other demographic factors, such as age group, gender, nationality, region, marital status, and student status, did not show statistically significant associations with sleep sufficiency (Table 4).

**Table 4:** Bivariate analysis of demographic characteristics, gaming behaviors, and their impact on sleep deficiency p-values are considered statistically significant if less than 0.05.

Variable	Category	Insufficient sleep		p-value
		Frequency	Percent	
Age group (years)	Under 20	45	49.5%	0.35
	20–22	53	60.2%	
	23–25	66	53.7%	
Gender	Male	80	58.4%	0.19
	Female	84	50.9%	
Nationality	Saudi	154	53.7%	0.32
	Non-Saudi	10	66.7%	
Region	Eastern Province	146	55.5%	0.09
	Western Province	6	60.0%	
	Northern Province	2	18.2%	
	Central Province	9	64.3%	
	Southern Province	1	25.0%	
Marital status	Single	96	49.5%	0.07
	Married	57	62.0%	
	Divorced	11	68.8%	
Student status	No	53	53.0%	0.75
	Yes	111	55.0%	
Body mass index category	Underweight	2	18.2%	<0.01
	Normal weight	73	53.3%	
	Overweight	39	67.2%	
	Obese	7	29.2%	
Daily gaming duration	Less than one hour	69	63.3%	0.02
	One to three hours	57	54.3%	
	More than three hours	38	43.2%	
Timing of gaming sessions	Before completing tasks	31	86.1%	<0.01
	After completing tasks	41	53.9%	
	During break time	47	47.0%	
	Timing does not matter	45	50.0%	
Gaming type preference	Offline	22	41.5%	0.04
	Online	142	57.0%	
Variation in gaming time	More on weekdays	55	46.2%	0.02
	More on weekends	109	59.6%	

Multivariable logistic regression analysis

The multivariable logistic regression analysis provided insights into the factors associated with sleep insufficiency, revealing significant associations and trends (Table 5). Gender showed a borderline significant association, with females having half the odds of sleep insufficiency compared to males (OR = 0.5, 95% CI (0.3-1.0), p = 0.05). Marital status highlighted that divorced participants had notably higher odds of experiencing sleep insufficiency compared to singles, although this was marginally nonsignificant (OR = 4.3, 95% CI (0.9-19.3), p = 0.06).

In terms of body mass index categories, overweight participants were significantly more likely to experience sleep insufficiency compared to the underweight reference category (OR = 7.7, 95% CI (1.4-42.3), p = 0.02). However, the normal weight and obese categories did not show a statistically significant difference.

Gaming habits also demonstrated significant relationships with sleep insufficiency. Participants gaming after completing tasks (OR = 0.2, 95% CI (0.1-0.9), p = 0.04) or during break time (OR = 0.2, 95% CI (0.1-0.7), p = 0.02) had significantly lower odds of sleep insufficiency compared to those gaming before completing tasks. Variation in gaming time indicated that those gaming more on weekends had twice the odds of experiencing sleep insufficiency than those gaming more on weekdays (OR = 2.0, 95% CI (1.0-3.8), p = 0.05).

**Table 5:** Multivariable logistic regression analysis of factors associated with sleep insufficiency p-values are considered statistically significant if less than 0.05.

Variable	Category	Logistic regression		p-value
		OR	(95% CI)	
Gender	Male	Reference category		
	Female	0.5	(0.3–1.0)	0.05
Marital status	Single	Reference category		
	Married	1.5	(0.8–3.1)	0.24
	Divorced	4.3	(0.9–19.3)	0.06
Body mass index category	Underweight	Reference category		
	Normal weight	4.5	(8.7–23.8)	0.07
	Overweight	7.7	(1.4–42.3)	0.02
	Obese	1.2	(0.2–7.9)	0.84
Daily gaming duration	Less than one hour	Reference category		
	One to three hours	0.9	(0.4–1.7)	0.67
	More than three hours	0.7	(0.3–1.6)	0.46
Timing of gaming sessions	Before completing tasks	Reference category		
	After completing tasks	0.2	(0.1–0.9)	0.04
	During break time	0.2	(0.1–0.7)	0.02
	Timing does not matter	0.3	(0.1–1.1)	0.06
Gaming type preference	Offline	Reference category		
	Online	1.3	(0.6–2.9)	0.52
Variation in gaming time	More on weekdays	Reference category		
	More on weekends	2	(1.0–3.8)	0.05

## Discussion

The findings from this study provide a comprehensive understanding of the intricate relationship between video game usage, sleep patterns, and health outcomes among young adults in the Al-Ahsa region of Saudi Arabia. The results reveal a significant association between extended video game play and disrupted sleep patterns, aligning with the initial hypothesis and echoing the concerns raised in the literature about the potential adverse effects of excessive gaming on physical and psychological health.

The study’s analysis indicates that participants engaging in prolonged periods of video game play, especially before bedtime, are more likely to experience difficulties initiating sleep and maintaining sleep quality. This association supports previous research suggesting that the stimulating effects of video games can delay sleep onset and reduce sleep duration, potentially through the suppression of melatonin production due to exposure to screen light [8]. Moreover, the preference for online gaming, which often involves interactive and competitive elements, may further exacerbate these effects by increasing psychological arousal before sleep [9].

Concerning health outcomes, the link between disrupted sleep patterns and an increased prevalence of health issues such as obesity, hypertension, and insulin resistance among gamers is particularly noteworthy. These findings are consistent with the broader body of evidence indicating that poor sleep quality and duration are significant risk factors for these conditions [10]. The study’s data on body mass index categories further illuminate this relationship, showing a higher frequency of sleep insufficiency among overweight participants, which underscores the bidirectional relationship between sleep and obesity.

The study’s focus on the Al-Ahsa region adds valuable insights into how cultural and regional factors might influence the relationship between video gaming, sleep, and health. The high prevalence of video gaming among young adults in this region, as predicted in the study’s hypothesis, points to the need for culturally tailored interventions that address both the behavioral aspects of gaming and the underlying social and environmental factors that facilitate excessive gaming habits.

These findings suggest several important implications for public health strategies and policy development [11,12]. There is a clear need for public health initiatives that educate young adults about the potential risks associated with excessive video gaming, particularly regarding sleep hygiene and health. Schools, universities, and community organizations in Al-Ahsa and similar regions could play a crucial role in promoting healthier digital media usage patterns through workshops, awareness campaigns, and counseling services.

Additionally, the study highlights the potential benefits of incorporating sleep education into preventive health care for young adults, emphasizing the importance of maintaining a balanced relationship with digital media. Policymakers may also consider regulations that encourage video game developers to include features that minimize the potential for sleep disruption, such as reminders to take breaks or warnings about playing close to bedtime [13-15].

While the study provides significant insights, it is not without limitations. The cross-sectional design limits the ability to infer causality between video gaming, sleep patterns, and health outcomes. Future research could benefit from longitudinal studies to better understand the causal relationships and the long-term effects of video gaming habits. Additionally, exploring the role of specific types of video games and gaming contexts (e.g., competitive vs. casual gaming) could offer a more nuanced understanding of how different gaming experiences affect sleep and health.

## Conclusions

This study elucidates the significant impact of video game usage on sleep patterns and subsequent health outcomes among young adults in the Al-Ahsa region of Saudi Arabia. The findings corroborate the hypothesis that excessive gaming can lead to sleep disturbances, which, in turn, may precipitate various health issues, including obesity, hypertension, and insulin resistance. These insights highlight the necessity for targeted public health interventions and educational programs aimed at promoting healthier gaming behaviors and sleep hygiene practices among young adults. Furthermore, the study calls for the development of policies that encourage responsible gaming habits, emphasizing the critical need for a balanced approach to video game engagement to safeguard the well-being of this vulnerable population. Future research should expand upon these findings through longitudinal studies to explore the long-term effects of video gaming on health and well-being, providing a more comprehensive understanding of this multifaceted issue.
